# Changes to Endemic Respiratory Virus Circulation and Testing Before, During, and After the COVID-19 Pandemic

**DOI:** 10.1093/ofid/ofaf493

**Published:** 2025-09-26

**Authors:** Kim El-Haddad, Wei Liu, Patrick Burke, Hannah Wang, Frank P Esper

**Affiliations:** Department of Pediatrics, Center for Pediatric Infectious Disease, Cleveland Clinic Children's, Cleveland, Ohio, USA; Department of Quantitative Health Sciences, Cleveland Clinic, Cleveland, Ohio, USA; Department of Infection Prevention, Cleveland Clinic, Cleveland, Ohio, USA; Department of Pathology and Laboratory Medicine, Cleveland Clinic, Cleveland, Ohio, USA; Cleveland Clinic Lerner College of Medicine of Case Western Reserve University, Cleveland, Ohio, USA; Department of Pediatrics, Center for Pediatric Infectious Disease, Cleveland Clinic Children's, Cleveland, Ohio, USA; Cleveland Clinic Lerner College of Medicine of Case Western Reserve University, Cleveland, Ohio, USA

**Keywords:** SARS-CoV-2 pandemic, respiratory virus seasonality, influenza A and RSV, viral displacement, respiratory virus circulation

## Abstract

**Background:**

The SARS-CoV-2 pandemic and resulting countermeasures significantly disrupted the circulation of many endemic respiratory viruses. While most viruses experienced sharp declines immediately following the pandemic onset, recovery dynamics varied among species. We hypothesize that the degree of displacement in virus circulation and the time required to return to prepandemic patterns are influenced by the circulation overlap with SARS-CoV-2.

**Methods:**

Respiratory virus testing data from nasopharyngeal specimens (2015 through May 2024) were analyzed for 6 respiratory viruses grouped into 3 seasonality patterns: wintertime (influenza A, respiratory syncytial virus), springtime (human metapneumovirus, parainfluenza 3), and year-round (adenovirus, rhinovirus/enterovirus). Prepandemic and post–SARS-CoV-2 emergence trends in positivity and peak displacement were assessed and compared in interrupted time series, wavelet, and median regression analyses.

**Results:**

Marked disruptions in respiratory virus patterns occurred following the local spread of SARS-CoV-2. Winter viruses (influenza A and respiratory syncytial virus) showed significant declines in positivity during the postemergence period, with protracted resurgence thereafter. Spring viruses (parainfluenza 3 and human metapneumovirus) and year-round viruses (rhinovirus/enterovirus and adenovirus) were resilient, quickly returning to prepandemic positivity. Interrupted series and wavelet analyses revealed altered seasonality for winter viruses, with influenza A transitioning to a semiannual pattern before normalizing in 2024. Median peak displacement for spring viruses normalized within 2 seasons postemergence, while winter viruses had more pronounced shifts in seasonal timing.

**Conclusions:**

The COVID-19 pandemic and resulting countermeasures disproportionately affected winter-dominant viruses, delaying their recovery to prepandemic patterns in Cleveland, Ohio. Spring and year-round viruses experienced milder deviations and have largely normalized. These findings provide key insights for future pandemic preparedness.

**Article Summary:**

The COVID-19 pandemic disproportionately affected endemic viruses whose circulation closely overlaps with peak SARS-CoV-2 circulation. Winter-dominant viruses had more severe and prolonged alterations while spring and year-round viruses experienced milder changes and recovered more quickly.

Respiratory viruses have expected patterns of circulation at reasonably predictable intervals despite mild annual variances [1]. Factors contributing to a virus's seasonal preferences and periodicity remain poorly understood but may include environmental conditions (temperature, humidity), host behaviors (indoor activities, travel), and characteristics unique to the virus (genome stability, mode of transmission) [2]. Additionally, circulation patterns differ substantially between temperate and tropical regions [3]. In temperate climates of North America, most respiratory viruses cluster in 3 seasonal patterns (fall, winter, and spring). Few respiratory viruses preferentially circulate during the summer, but a handful are detected year-round without specific seasonal preference. In the northern latitudes of the United States, parainfluenza 1 and 2 alternate in a biennial circulation and typically peak in the fall. Respiratory syncytial virus (RSV), influenza A (FLUA), and endemic coronaviruses (OC43, HKU1, 229E, NL63) predominantly circulate during the winter months, often peaking December through February [4]. Human metapneumovirus (HMPV) and parainfluenza 3 (PIV3) typically circulate during the springtime, peaking from March through June [5, 6]. Human rhinoviruses (RHINO), parainfluenza 4, and adenovirus (ADENO) are commonly detected year-round without seasonal bias. Enteroviruses (ENTERO), while detected year-round, has a late summer/early fall preference [7, 8]. The SARS-CoV-2 virus was introduced into the United States in January 2020 and characterized as a pandemic on 11 March 2020 [9]. Since then, SARS-CoV-2 has maintained semiannual peaks with winter (commonly in January and February) and summer (July and August) [10].

Following the onset of the SARS-CoV-2 pandemic and subsequent community countermeasures (eg, physical distancing and mask wearing), most endemic respiratory viruses became historically low [11, 12]. In 2021, with the relaxation of many of these public health practices, endemic respiratory viral infections returned. However, circulation patterns were substantially out of phase with prepandemic norms. The most notable was recurrence of RSV circulation in late summer of 2021 with prolonged circulation and higher disease severity straining many unprepared health care systems [13, 14]. Also, detection of influenza B decreased globally with extinction of the Yamagata (B/Yam) lineage [15].

Disruptions to endemic virus circulation have been reported following previous pandemics. The H1N1 influenza pandemic of 2009 led to a decline in seasonal viruses in all age groups [16]. In 2003, the SARS-CoV-1 pandemic was associated with a marked reduction in the circulation of influenza and RSV [16, 17]. Still, the effects from both these pandemics were short-lived with near normalization of endemic virus circulation patterns returning within a year. In contrast, SARS-CoV-2 resulted in worldwide alterations over multiple seasons affecting multiple respiratory viruses. However, the extent and duration of displacement was not uniform across virus species.

We hypothesize that the degree of change in endemic viral positivity and the time to return to prepandemic patterns are associated with the degree of circulation overlap with SARS-CoV-2 in the community. Here we analyze circulation recovery of endemic viruses post–SARS-CoV-2 emergence based on their seasonality in a temperate climate.

## METHODS

### Database Construction

The Cleveland Clinic is a large tertiary hospital system based in Cleveland, Ohio, that includes 15 regional hospitals and >250 outpatient locations with 11.1 million patient encounters annually, serving nearly half of all households within the community [18]. Results were tabulated from respiratory pathogen tests from children and adults submitted to the Cleveland Clinic from 29 December 2014 (2015 International Organization for Standardization [ISO] week 1) through 5 May 2024 (2024 ISO week 18). Only laboratory-based nucleic acid amplification test results originating from nasopharyngeal swabs were included. Results originating from antigen testing or viral culture (representing only 0.12% of all virus tests during the study period) were excluded from the result database. Results originated from patients tested in outpatient clinics, inpatient wards, and emergency departments at the discretion of the on-service physicians. Clinical testing platforms and assays varied throughout the study period (Supplementary Methods, Supplementary Table 1). Viral targets used in database construction include RSV A/B, parainfluenza virus 1/2/3/4, HMPV, RHINO/ENTERO, ADENO, FLUA types (H1/H3/H1pdm09), influenza B, human bocavirus, and human coronaviruses OC43/229E/NL63/HKU1 and SARS-CoV-2.

Following database construction, duplicate results of patients with multiple samples tested <30 days apart were removed from analysis. Data for RSV A and RSV B were collectively grouped into RSV. Similarly, FLUA data typed H1, H3, and pdmH1, as well as those not typed, were placed into the FLUA group. The COVID-19 pandemic onset date was defined as 9 March 2020 (first SARS-CoV-2–positive result in Cleveland Clinic Foundation database), and the prepandemic period was defined as 29 December 2014 to 8 March 2020. Time following the pandemic onset was divided into the immediate postemergence period (9 March–10 May 2020; period of nationwide emergency), when the study region experienced state-of-emergency orders and endemic virus testing was limited (supplementary materials), and the post–SARS-CoV-2 emergence period (11 May 2020–5 May 2024) [19].

Viruses were grouped into 3 seasonal circulation patterns based on prepandemic patterns: wintertime group, FLUA and RSV; springtime group, HMPV and PIV3; and year-round group, RHINO/ENTERO and ADENO. Human coronaviruses (HKU1, OC43, 229E, NL63) and human bocavirus, as well as parainfluenza types 1, 2, and 4, were not used in analysis due to inadequate prepandemic test volumes needed for analysis. Influenza B positivity significantly decreased in the postpandemic period (6.1% prepandemic vs 0.18% postemergence, *P* < .0001) and therefore was not included in the analysis.

### Statistical Analysis

Interrupted time series (ITS) analysis was used to evaluate trends in percentage positivity before and after the pandemic onset and to assess the influence of the outbreak on virus positivity [20]. The percentages of reported positive cases in the corresponding tested samples were sequentially summarized quarter-yearly in the study period. Time division for quarters were constructed to have 9 March 2020 (regional SARS-CoV-2 introduction) as a beginning of a quarter. The first 4 quarters following introduction were excluded from ITS analysis (time of low virus circulation and testing) to prevent skewing of results. Autocorrelation was assessed up to the 12th order and backward selected for final model (significance criterion for removal = .05). The prepandemic or postemergence slopes indicate yearly increases or decreases in the percentage of positive cases over a specific study period; the immediate impact indicates the change in the percentage of positive cases following SARS-CoV-2 introduction.

To assess virus periodic cycle over time, analysis of univariate times series via the Morlet wavelet was used [21]. Wavelet analysis is a mathematical technique used to analyze and decompose time series data in time and periodicity domains simultaneously, and it reveals how the different periodic components of a particular time series change over time. The method was selected as it does not require stationary data and is more capable when data show unusual peaks, interruptions, or smooth changes in periodicity. Weekly positive cases for each virus were included for analysis, and all time series were logarithm transformed (after adding a constant of 10) to become more sinusoidal. Weeks were defined from Monday to Sunday so that each week either fell in the prepandemic or post–SARS-CoV-2 emergence period. The wavelet power spectrum was used to visualize the power of periodic components over time, with red indicating high power and blue low power. White contour lines were added to show regions of significant power based on 1000 simulations. The cone of influence reflects loss in statistical power near the start and end of the series; the region under the parabola without the white shade is free from these edge effects.

To assess peak shifts in winter and spring viruses between the prepandemic period and subsequent cycling years with observed peaks, a median regression was employed with ISO weeks of virus positivity as the outcome and categorical time as the predictor (prepandemic, outbreak–2021, 2021–2022, 2022–2023, and 2023–2024). The regression parameters were estimated by either the simplex or interior point algorithm, depending on the number of observations in the model. Confidence intervals for the differences in medians between time groups were calculated per the Bofinger sparsity method, assuming the error term to be independent and identically distributed. Each virus's cycling year was defined by its unique seasonality patterns, ensuring that no major peaks were split across 2 cycling years. ISO weeks were rescaled before modeling, with the first week of a cycling year assigned the smallest index and the last week the largest. Because ISO week 53 was rare during the study period, it was assigned the same index as week 52.

All tests were 2-tailed and performed at an overall significance level of .05. Analysis utilized SAS version 9.4, R version 4.4.1, and Python version 3.10 with several open-source packages (Supplementary Methods) for analyses and visualizations. The ITS models were performed with the AUTOREG procedure in SAS/ETS. The wavelet analysis was performed in the WaveletComp package (version 1.1) in R.

## RESULTS

Of 3 006 962 respiratory virus results from 1 402 528 tests occurring from 29 December 2014 (2015 ISO week 1) through 5 May 2024 (2024 ISO week 18) at the Cleveland Clinic, 1 203 405 (40.0%) results were used in the analysis database (Table 1, Supplementary Table 1). This included 326 578 children (<18 years, 27.1%) and 876 827 adults (≥18 years, 72.9%). The remaining 1 803 557 (60.0%) sample results involved viruses not used in the analysis.

**Table 1.** Prepandemic and Postemergence Cumulative Respiratory Viral Results

	Overall (N = 1 203 405)		Prepandemic: 29 Dec 2014–8 Mar 2020 (n = 308 567)		Immediate Postemergence: 9 Mar 2020–10 May 2020 (n = 22 173)		Postemergence: 11 May 2020–5 May 2024 (n = 872 665)		
Virus	No.	Positive (%)	No.	Positive (%)	No.	Positive (%)	No.	Positive (%)	*P* Value^a^
Influenza A	539 562	44 945 (8.3)	158 252	23 239 (14.7)	9894	702 (7.1)	371 416	21 004 (5.7)^b^	<.0001
Respiratory syncytial virus	438 783	21 145 (4.8)	69 679	7057 (10.1)	8226	91 (1.1)	360 878	13 997 (3.9)^b^	<.0001
Parainfluenza virus 3	55 412	1307 (2.4)	20 128	501 (2.5)	989	4 (0.4)	34 295	802 (2.3)	.0001
Human metapneumovirus	55 404	1905 (3.4)	20 132	782 (3.9)	992	45 (4.5)	34 280	1078 (3.1)^b^	<.0001
Adenovirus	56 860	1765 (3.1)	20 123	591 (2.9)	1033	17 (1.6)	35 704	1157 (3.2)	.0034
Rhinovirus/enterovirus	57 384	8488 (14.8)	20 253	3228 (15.9)	1039	79 (7.6)	36 092	5181 (14.4)^b^	<.0001

^a^Pearson χ^2^ test. Note that each *P* < .05.

^b^Significantly different between prepandemic and postemergence after applying Bonferroni correction for multiple comparisons.

### Winter-Dominant Viruses (FLUA, RSV)

**Figure 1.**
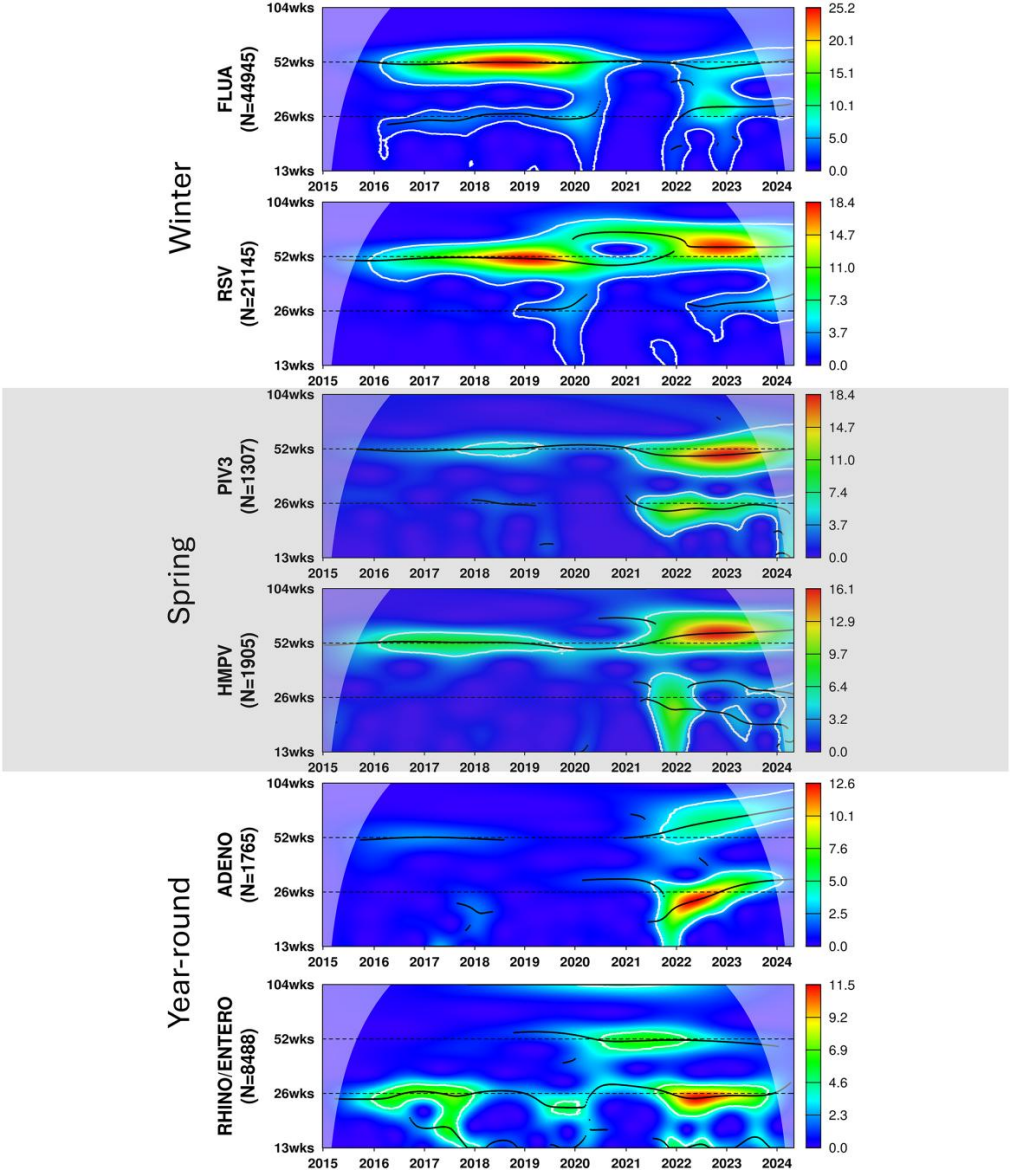
Wavelet analysis shows prepandemic to post–SARS-COV-2 emergence changes in endemic virus circulation periodicity. Weekly positive cases for each virus were analyzed by Morlet wavelet analysis, which identifies changes in periodicity over time. The wavelet power spectrum visualizes the strength of periodic pattern over time, with red indicating high power and blue indicating low power (horizontal scale left of figure). The white contour lines outline regions where power is statistically significant at the 5% level, based on 1000 simulations. The black dotted lines mark the peaks of periodic fluctuations in the spectrum. The cone of influence, represented by the curved boundary, indicates areas near the start and end of the time series where statistical power is reduced due to edge effects. Regions under the parabola without the white shade are unaffected by these edge effects. Abbreviations: ADENO, adenovirus; ENTERO, enterovirus; FLUA, influenza A; HMPV, human metapneumovirus; PIV3, parainfluenza 3; RHINO, rhinovirus; RSV, respiratory syncytial virus.

**Figure 2.**
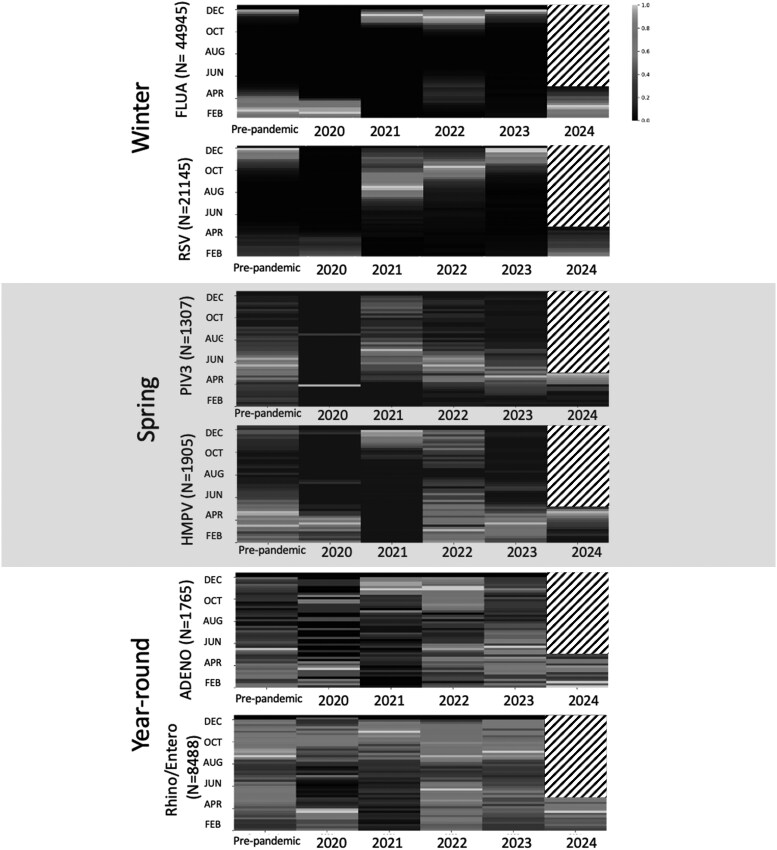
Circulation heat map of endemic viruses. Normalized heat maps of annual virus circulation by International Organization for Standardization weeks (months displayed for ease of reading): prepandemic (29 December 2014–8 March 2020), immediate post–SARS-CoV-2 emergence (9 March–5 May 2020), and postemergence (6 May 2020–5 May 2024). Number of total viral tests during study period are shown for each virus species. Prepandemic data represent average circulation distribution for 2015 to 2019. Normalized values are displayed, with black representing weeks with low circulation and white with high circulation. Study period ended May 2024, with all dates thereafter appearing hatched. Abbreviations: ADENO, adenovirus; ENTERO, enterovirus; FLUA, influenza A; HMPV, human metapneumovirus; PIV3, parainfluenza 3; RHINO, rhinovirus; RSV, respiratory syncytial virus.

Prepandemic winter viruses exhibited distinct single peaks in circulation, with a consistent 52-week annual cycle (Figure 1). Circulation predominated during December through February with median peaks at ISO weeks 6 and 52 for FLUA and RSV, respectively (Figure 2). FLUA and RSV showed peak activity more temporally aligned with SARS-CoV-2, in contrast to spring-dominant viruses (Table 2, Supplementary Table 2).

**Table 2.** Overlap Between Peak Positivity of Endemic Viruses (Prepandemic) and SARS-CoV-2 (Since Outbreak)

Virus^a^	Median Difference^b^ (95% CI)	*P* Value
Influenza A	5.0 (4.9, 5.1)	<.0001
Respiratory syncytial virus	−1.0 (−1.2, −.8)	<.0001
Parainfluenza virus 3	19.0 (18.3, 19.7)	<.0001
Human metapneumovirus	10.0 (9.4, 10.6)	<.0001

Prepandemic: 29 December 2014–8 March 2020. Since outbreak: 9 March 2020–5 May 2024.

^a^Reference: SARS-CoV-2.

^b^The median difference in International Organization for Standardization weeks of positivity as compared with SARS-CoV-2 indicates the extent of distribution overlap, with smaller differences representing a higher degree of overlap.

#### Influenza A

**Figure 3.**
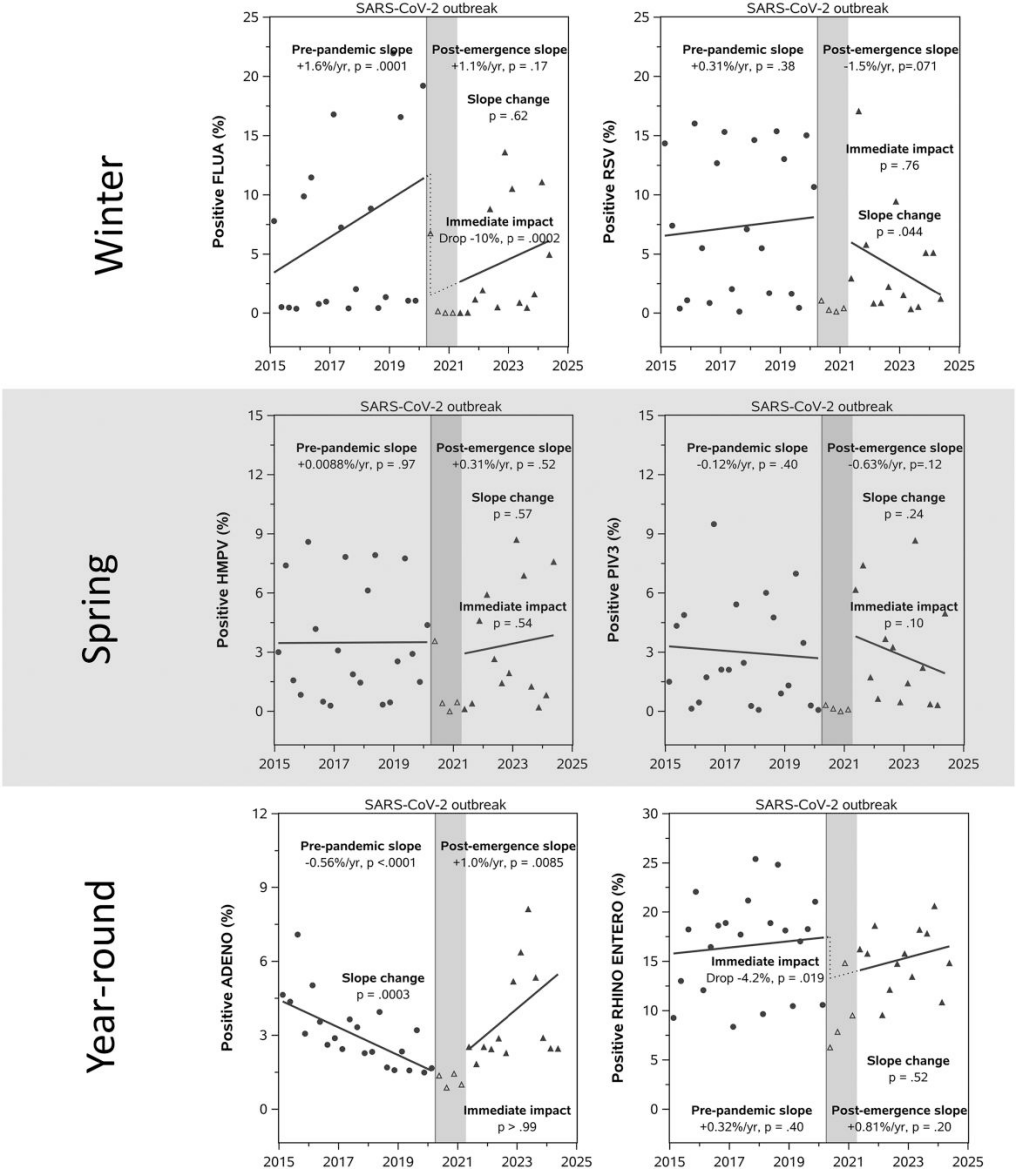
Interrupted time series analysis of endemic virus circulation (2015–2024). Percentage positivity of winter, spring, and year-round viruses. Each scatter point represents percentage positivity in a quarter, with filled circles for prepandemic and filled triangles for postemergence data. Segmented regression lines were fitted via interrupted time series analysis. The first 4 quarters (open triangle points in the gray shaded area) following SARS-CoV-2 introduction were excluded from analysis due to low virus circulation to prevent skewing of results. The prepandemic or postemergence slope indicates a yearly increase or decrease in the percentage of positive cases over a specific study period; the immediate impact indicates the sudden change in the percentage of positive cases immediately following SARS-CoV-2 introduction. Abbreviations: ADENO, adenovirus; ENTERO, enterovirus; FLUA, influenza A; HMPV, human metapneumovirus; PIV3, parainfluenza 3; RHINO, rhinovirus; RSV, respiratory syncytial virus.

FLUA was the most predominant endemic respiratory virus detected, with 8.3% (44 945/539 562) positivity over the study period (Table 1). Overall, FLUA positivity declined by 9.0% (95% CI, 8.8%–9.2%) in the postemergence period as compared with prepandemic (Supplementary Table 3). ITS analysis showed that FLUA positivity increased by 1.6% (95% CI, 0.87%–2.3%; *P* = .0001) per year prepandemic, dropped immediately by 10% (95% CI, 5.3%–15%; *P* = .0002) following the pandemic onset, and trended upward thereafter (+1.1% per year, *P* = .17; Figure 3, Supplementary Table 4).

**Figure 4.**
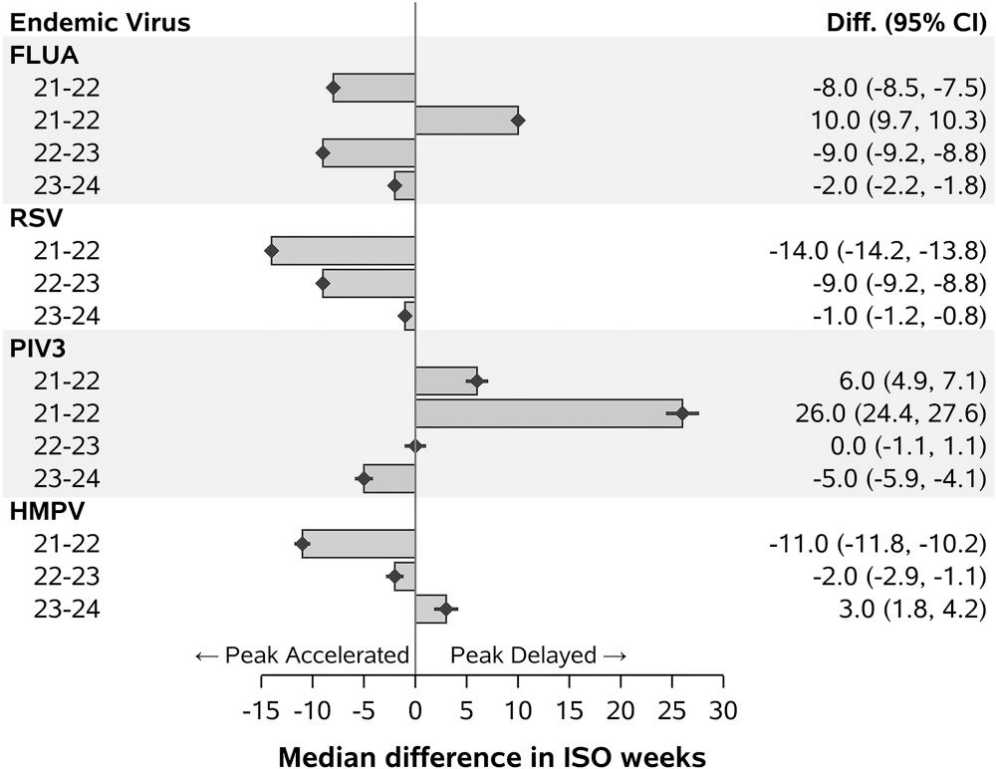
Median peak displacement of winter and spring viruses. The median peak displacement for viruses (FLUA, RSV, PIV3, and HMPV) over 3 post–SARS-CoV-2 emergence seasons (2021–2024) is represented as the difference in median ISO weeks as compared with the prepandemic average. Positive values indicate a delay in peak timing, while negative values indicate that peaks occurred earlier than usual. In 2022, FLUA and PIV3 exhibited prominent semiannual patterns resulting in 2 measurement points. Abbreviations: FLUA, influenza A; HMPV, human metapneumovirus; ISO, International Organization for Standardization; PIV3, parainfluenza 3; RSV, respiratory syncytial virus.

Wavelet analysis demonstrated a shift of FLUA's classic winter seasonality after the pandemic, with the emergence of semiannual (26-week) cycles since 2022 (Figure 1, Supplementary Figure 1). From 2022 to 2023, the semiannual pattern dominated, followed by a partial return to an annual pattern. Median peak displacement analysis revealed an 8-week earlier peak (95% CI, 7.5–8.5) from 2021 to 2022, with normalization to a −2.0-week shift (95% CI, −2.2 to −1.8) by 2023 to 2024 (Figure 4).

#### Respiratory Syncytial Virus

RSV positivity was the second-highest occurrence among respiratory viruses, detected in 4.8% of specimens (21 145/438 783; Table 1). Overall, RSV showed a 6.2% (95% CI, 6.0%–6.5%) decline in positivity postemergence (Supplementary Table 3). ITS analysis showed a postemergence decline of 1.5% per year (95% CI, −0.13% to 3.1%; *P* = .071), with a significantly different trend as compared with prepandemic (*P* = .044; Figure 3, Supplementary Table 4).

Wavelet analysis revealed that RSV's prepandemic annual cycle was disrupted after 2020, with a lengthened peak cycle over 52 weeks (Figure 1, Supplementary Figure 1). RSV showed the most prominent peak displacement in 2021 to 2022, with peaks occurring 14.0 weeks (95% CI, 13.8–14.2) earlier than the prepandemic average (Figure 4). Peak RSV seasonality slowly normalized over subsequent seasons, reaching near prepandemic timing by 2023 to 2024.

### Spring-Dominant Viruses (HMPV, PIV3)

Before the pandemic, spring viruses followed an annual cycle with consistent 52-week periodicity (Figure 1). Circulation predominated during March through June (Figure 2). Median peak activity occurred at ISO weeks 11 and 20 for HMPV and PIV3, respectively, which poorly overlapped with SARS-COV-2 circulation postemergence (Supplementary Figure 1, Table 2, Supplementary Table 2).

#### Parainfluenza Virus 3

PIV3 positivity showed a marked decline to 0.4% in the immediate postemergence period, followed by a recovery to 2.3% thereafter (Table 1). ITS analysis showed that percentage positivity did not significantly change between the prepandemic and postemergence periods (Figure 3, Supplementary Table 4). Wavelet analysis demonstrated that PIV3's annual seasonality was disrupted during the 2020 SARS-CoV-2 outbreak, with a recovery beginning in 2021 and the emergence of a concurrent semiannual cycle (Figure 1, Supplementary Figure 1). Median peak displacement analysis showed a 6-week delay (95% CI, 4.9–7.1) in 2021 to 2022, returning to baseline timing by 2022 to 2023 (Figure 4).

#### Human Metapneumovirus

HMPV positivity remained relatively stable, maintaining a 4.5% positivity rate immediately postemergence and 3.1% postemergence as compared with 3.9% prepandemic (Table 1). ITS analysis showed that percentage positivity did not significantly change over time in the prepandemic and postemergence periods (Figure 3, Supplementary Table 4). Wavelet analysis revealed that HMPV's prepandemic annual cycle was disrupted after 2020, with a recovery beginning in 2021 and a slightly extended peak interval over 52 weeks; a transient semiannual pattern emerged since late 2021 and gradually faded in 2023 (Figure 1, Supplementary Figure 1). Median peak displacement analysis showed that HMPV peaks occurred 11 weeks (95% CI, 10.2–11.8) earlier during the 2021–2022 season and normalized to 2-week advancement (95% CI, 1.1–2.9) by 2022 to 2023 (Figure 4).

### Year-round Viruses (RHINO/ENTERO, ADENO)

Before the pandemic, the year-round viruses exhibited irregular, noise-like spikes, with no significant seasonality in ADENO and a slight semiannual cycle in RHINO/ENTERO during partial years (Figure 1, Supplementary Figure 1).

#### Rhinovirus/Enterovirus

RHINO/ENTERO positivity demonstrated resilience, maintaining 14.4% postemergence positivity as compared with 15.9% prepandemic (Table 1). ITS analysis indicated no significant prepandemic trend (*P* = .40), although there was an immediate drop postemergence of 4.2% (95% CI, 0.75%–7.6%; *P* = .019), which quickly stabilized (Figure 3, Supplementary Table 3). Wavelet analysis showed that RHINO/ENTERO exhibited more consistent semiannual cycles post–SARS-CoV-2 emergence, developing since late 2021 (Figure 1, Supplementary Figure 1).

#### Adenovirus

ADENO showed strong resilience. After an initial drop to 1.6% positivity immediately postemergence, it recovered to 3.2%, slightly higher than its prepandemic rate of 2.9% (Table 1). ITS analysis indicated that ADENO positivity, which had been decreasing by 0.56% per year prepandemic (95% CI, 0.40%–0.72%; *P* < .0001), reversed to an increasing trend of +1.0% per year postemergence (95% CI, 0.28%–1.7%; *P* = .0085; for trend change, *P* = .0003; Figure 3, Supplementary Table 4). Wavelet analysis showed an approximate semiannual signal for ADENO following the emergence of SARS-CoV-2 (Figure 1, Supplementary Figure 1).

## DISCUSSION

Our study describes and quantifies the influence of the COVID-19 pandemic on endemic respiratory virus circulation patterns in Cleveland, Ohio. Winter-dominant viruses (FLUA, RSV) that had substantial overlap with pandemic peaks experienced sharper declines in positivity and more prolonged displacement of peak circulation. This reduction likely reflects the broader impact on respiratory virus transmission caused by widespread community mitigation measures such as masking, physical distancing, and school closures implemented in response to active COVID-19 circulation. Conversely, spring-dominant viruses such as HMPV and PIV3 had minimal changes in positivity and demonstrated limited seasonal displacement. Year-round viruses such as RHINO/ENTERO and ADENO were more resilient, maintaining activity post–SARS-CoV-2 emergence and quickly returning to prepandemic patterns within a year. Our community's circulation data align closely with the data presented in the Centers for Disease Control and Prevention's National Respiratory and Enteric Virus Surveillance System [10]. Unlike the aggregate data reported in this system, our dataset provides a more granular, community-level view and allows for the removal of duplicative tests from the same patient, offering a more accurate representation of virus circulation patterns.

Most respiratory viruses were absent following the initial pandemic waves in many countries [22]. The mechanisms driving the circulation of respiratory pathogens have been a subject of ongoing investigation, with environmental factors and human behavioral changes recognized as primary contributors [23]. Following SARS-CoV-2 emergence, the Ohio Department of Health implemented substantial public health measures to mitigate the spread of the virus (supplementary materials) [19, 24]. Winter-dominant viruses, specifically influenza and RSV, rely on the movement of infections between regions to sustain their circulation and maintain global seasonal patterns. Many respiratory viruses are known to circulate between the Northern and Southern Hemispheres, with seasonal peaks alternating between winter months in each hemisphere [25, 26]. The interruption of travel significantly reduced the importation of these viruses from endemic regions, disrupting their transmission cycle and leading to an absence or sharp decline in their activity during the pandemic [27]. This underscores the impact of travel restrictions on virus dynamics [28]. Also, infection with a new pandemic virus can lead to indirect suppression of other respiratory viruses through mechanisms such as nonspecific priming of the immune response. Nonspecific T-cell activation and cytokine (interferon) release may confer temporary immunity to the host and reduce host susceptibility to subsequent viral challenges [29]. As such, the reduction in exposure to common respiratory viruses during the pandemic likely contributed to an accumulation of susceptible individuals, leading to an altered incidence of disease once endemic virus circulation was restored [30]. It is thought that this immunity gap predisposed the population to larger, more severe outbreaks demonstrated by the RSV resurgence in 2022 [31]. Nirsevimab and the RSV vaccines were Food and Drug Administration approved in 2023. However, their use was limited in the first season of use and unlikely to have an impact on our RSV analysis [32, 33].

Spring viruses may have been less affected as their peak circulation occurred after nonspecific immunity waned. Spring circulating viruses also peaked between the semiannual SARS-CoV-2 peaks when nonpharmaceutical intervention (eg, masking) may have lessened. Year-round viruses such as RHINOs and ADENOs displayed minimal disturbance, and their transmission occurred consistently throughout the pandemic. The apparent “year-round” distribution of certain viruses may be attributed to the continuous circulation of their numerous individual genotypes. For example, the ADENO family includes >50 distinct types, while the ENTERO genus consists of an even greater number of members. This diversity may contribute to their sustained presence throughout the year, despite fluctuations in individual genotype prevalence. Here we analyzed ADENO and RHINO/ENTERO at the species level. If analysis is performed at the level of the genotype, some seasonality may be seen.

Our study had several limitations. First, our database was populated by patient results collected at the discretion of on-service physicians and therefore may lead to a selection bias for viruses associated with more severe disease. Additionally, we are unable to distinguish whether the data reflect inpatient or outpatient encounters, as these were combined in our dataset. However, we feel that Cleveland Clinic's extensive coverage within the region, with considerable use of molecular viral testing, provides the best measure of viral circulation in this community. Other potential factors, such as health care–seeking behavior and testing practices, were not included in this analysis. Human coronaviruses, human bocavirus, and influenza B, as well as parainfluenza types 1, 2, and 4, were excluded from analysis due to insufficient prepandemic test volumes or significant post–SARS-CoV-2 decline in positivity. Further study is warranted to evaluate the pandemic's effect on circulation of these viruses as well as bacterial pathogens (mycoplasma, pneumococcus) and gastroenteric ones (rotavirus, norovirus). Our analysis is derived from a single health care system within a temperate climate. Because the circulation of pathogens was significantly influenced by community-specific pandemic prevention measures, it limits the generalizability of our findings. Similar analysis should be performed in more tropical areas where viral circulation is more prolonged. Also, the observed shifts in virus circulation may have been skewed through changes in postemergence health care–seeking behavior [34]. Although behavior was highly influenced immediately after SARS-CoV-2 emergence, testing remained primarily targeted toward symptomatic individuals. Despite increased testing volume, the composition of the tested population between the prepandemic period and postemergence (especially 12 months postemergence) remained comparable. While we expect such impacts to be equally disruptive to all virus groups, we expanded the analysis to the 2023–2024 seasons where the medical landscape was more stable. To this point, we noted a significant drop in FLUA and RSV testing positivity post–SARS-CoV-2 emergence while spring and year-round viruses remained similar to prepandemic levels. These findings may be attributed to a “denominator effect” from elevated levels of SARS-CoV-2 testing incorporation into rapid testing with FLU A/B and RSV. Last, given that SARS-CoV-2 is evolving toward endemicity, it may continue to influence the spread of other viruses, suggesting that a full return to prepandemic seasonality may not occur [35].

## CONCLUSION

Overall, the data reveal considerable changes in respiratory virus detection after the onset of the SARS-CoV-2 pandemic, with more pronounced variations in viruses that closely cocirculated with SARS-CoV-2. In Cleveland, Ohio, winter virus circulation (FLUA, RSV) significantly shifted with slow reestablishment of prepandemic seasonal averages, while spring viruses (PIV3, HMPV) recovered to prepandemic patterns within 1 to 2 seasons and year-round viruses (ADENO, RHINO/ENTERO) quickly returned to baseline. Ours is one of the few studies providing a comprehensive overview of SARS-CoV-2's impact on winter, spring, and year-round viruses. Future surveillance and modeling efforts should account for the interplay of ecologic, immunologic, and societal factors to better predict and prepare for endemic virus–altered dynamics following future pandemics.

## Supplementary Material

ofaf493_Supplementary_Data

## Supplementary Data

Supplementary materials are available at *Open Forum Infectious Diseases* online. Consisting of data provided by the authors to benefit the reader, the posted materials are not copyedited and are the sole responsibility of the authors, so questions or comments should be addressed to the corresponding author.

## References

[1] Hawkes MT, Lee BE, Kanji JN, et al Seasonality of respiratory viruses at northern latitudes. JAMA Netw Open 2021; 4:e2124650. 34529066 10.1001/jamanetworkopen.2021.24650 PMC8446819

[2] Neumann G, Kawaoka Y. Seasonality of influenza and other respiratory viruses. EMBO Mol Med 2022; 14:e15352. 35157360 10.15252/emmm.202115352 PMC8988196

[3] Li Y, Reeves RM, Wang X, et al Global patterns in monthly activity of influenza virus, respiratory syncytial virus, parainfluenza virus, and metapneumovirus: a systematic analysis. Lancet Glob Health 2019; 7:e1031–45. 31303294 10.1016/S2214-109X(19)30264-5

[4] Lam TT, Tang JW, Lai FY, et al Comparative global epidemiology of influenza, respiratory syncytial and parainfluenza viruses, 2010–2015. J Infect 2019; 79:373–82. 31323249 10.1016/j.jinf.2019.07.008 PMC7112594

[5] Vinci A, Lee PJ, Krilov LR. Human metapneumovirus infection. Pediatr Rev 2018; 39:623–4. 30504257 10.1542/pir.2017-0213

[6] Fry AM, Curns AT, Harbour K, Hutwagner L, Holman RC, Anderson LJ. Seasonal trends of human parainfluenza viral infections: United States, 1990–2004. Clin Infect Dis 2006; 43:1016–22. 16983614 10.1086/507638

[7] Jacobs SE, Lamson DM, St George K, Walsh TJ. Human rhinoviruses. Clin Microbiol Rev 2013; 26:135–62. 23297263 10.1128/CMR.00077-12 PMC3553670

[8] Dela Cruz CS, Pasnick S, Gross JE, et al Adenovirus infection and outbreaks: what you need to know. Am J Respir Crit Care Med 2019; 199:P13–4. 30932693 10.1164/rccm.1997P13

[9] World Health Organization. Coronavirus disease (COVID-19) pandemic. Available at: https://www.who.int/europe/emergencies/situations/covid-19. Accessed 3 May 2025.

[10] Centers for Disease Control and Prevention . The National Respiratory and Enteric Virus Surveillance System (NREVSS): interactive dashboard. **2024**. Available at: https://www.cdc.gov/nrevss/php/dashboard/index.html. Accessed 16 January 2025.

[11] Piret J, Boivin G. Viral interference between respiratory viruses. Emerg Infect Dis 2022; 28:273–81. 35075991 10.3201/eid2802.211727 PMC8798701

[12] Chow EJ, Uyeki TM, Chu HY. The effects of the COVID-19 pandemic on community respiratory virus activity. Nat Rev Microbiol 2023; 21:195–210. 36253478 10.1038/s41579-022-00807-9 PMC9574826

[13] Hamid S, Winn A, Parikh R, et al Seasonality of respiratory syncytial virus—United States, 2017–2023. MMWR Morb Mortal Wkly Rep 2023; 72:355–61. 37022977 10.15585/mmwr.mm7214a1 PMC10078848

[14] Lee CY, Wu TH, Fang YP, et al Delayed respiratory syncytial virus outbreak in 2020 in Taiwan was correlated with two novel RSV-A genotype ON1 variants. Influenza Other Respir Viruses 2022; 16:511–20. 34913593 10.1111/irv.12951 PMC8983888

[15] Caini S, Meijer A, Nunes MC, et al Probable extinction of influenza B/Yamagata and its public health implications: a systematic literature review and assessment of global surveillance databases. Lancet Microbe 2024; 5:100851. 38729197 10.1016/S2666-5247(24)00066-1

[16] Yang L, Chan KH, Suen LKP, et al Impact of the 2009 H1N1 pandemic on age-specific epidemic curves of other respiratory viruses: a comparison of prepandemic, pandemic and post-pandemic periods in a subtropical city. PLoS One 2015; 10:e0125447. 25928217 10.1371/journal.pone.0125447 PMC4416050

[17] Lo JYC, Tsang THF, Leung Y-H, Yeung EYH, Wu T, Lim WWL. Respiratory infections during SARS outbreak, Hong Kong, 2003. Emerg Infect Dis 2005; 11:1738–41. 16318726 10.3201/eid1111.050729 PMC3367357

[18] Cleveland Clinic . Facts and figures. Available at: https://my.clevelandclinic.org/about/overview/who-we-are/facts-figures. Accessed 4 February 2025.

[19] Ideastream Public Media . Ohio's coronavirus pandemic: a timeline. Available at: https://www.ideastream.org/ohios-coronavirus-pandemic-a-timeline. Accessed 29 December 2024.

[20] Penfold RB, Zhang F. Use of interrupted time series analysis in evaluating health care quality improvements. Acad Pediatr 2013; 13:S38–44. 24268083 10.1016/j.acap.2013.08.002

[21] Grenfell BT, Bjørnstad ON, Kappey J. Travelling waves and spatial hierarchies in measles epidemics. Nature 2001; 414:716–23. 11742391 10.1038/414716a

[22] Stein RT, Zar HJ. RSV through the COVID-19 pandemic: burden, shifting epidemiology, and implications for the future. Pediatr Pulmonol 2023; 58:1631–9. 36811330 10.1002/ppul.26370

[23] Moriyama M, Hugentobler WJ, Iwasaki A. Seasonality of respiratory viral infections. Annu Rev Virol 2020; 7:83–101. 32196426 10.1146/annurev-virology-012420-022445

[24] Bricker Graydon . DeWine announces statewide facial covering requirement and travel advisory. **2020**. Available at: https://www.brickergraydon.com/insights/publications/DeWine-announces-statewide-facial-covering-requirement-and-travel-advisory. Accessed 26 April 2025.

[25] SARINET . Available at: https://sarinet.org/. Accessed 23 January 2025.

[26] SARINET . Seasonality of respiratory syncytial virus in Latin America and the Caribbean. Available at: https://sarinet.org/product/estacionalidad-de-virus-sincicial-respiratorio-en-latinoamerica-y-el-caribe/. Accessed 23 January 2025.

[27] Nolen LD, Seeman S, Bruden D, et al Impact of social distancing and travel restrictions on non-coronavirus disease 2019 (non–COVID-19) respiratory hospital admissions in young children in rural Alaska. Clin Infect Dis 2021; 72:2196–8. 32888007 10.1093/cid/ciaa1328 PMC7499549

[28] Huang QS, Turner N, Wood T, et al Impact of the COVID-19 related border restrictions on influenza and other common respiratory viral infections in New Zealand. Influenza Other Respir Viruses 2024; 18:e13247. 38350715 10.1111/irv.13247 PMC10864123

[29] Matera L, Manti S, Petrarca L, et al An overview on viral interference during SARS-CoV-2 pandemic. Front Pediatr 2023; 11:1308105. 38178911 10.3389/fped.2023.1308105 PMC10764478

[30] Ang HJ, Menegale F, Preziosi G, et al Reconstructing the impact of COVID-19 on the immunity gap and transmission of respiratory syncytial virus in Lombardy, Italy. eBioMedicine 2023; 95:104745. 37566927 10.1016/j.ebiom.2023.104745 PMC10432612

[31] Redlberger-Fritz M, Springer DN, Aberle SW, Camp JV, Aberle JH. Respiratory syncytial virus surge in 2022 caused by lineages already present before the COVID-19 pandemic. J Med Virol 2023; 95:e28830. 37282809 10.1002/jmv.28830

[32] Reses HE, Segovia G, Dubendris H, et al Coverage with influenza, respiratory syncytial virus, and COVID-19 vaccines among nursing home residents—National Healthcare Safety Network, United States, November 2024. MMWR Morb Mortal Wkly Rep 2024; 73:1052–7. 39570790 10.15585/mmwr.mm7346a2 PMC11581205

[33] Centers for Disease Control and Prevention . RSVVaxView: 2023–24 nirsevimab coverage (IIS), children 0 to 19 months. **2024**. Available at: https://www.cdc.gov/rsvvaxview/dashboard/2023-24-nirsevimab-coverage-jurisdiction.html. Accessed 4 February 2025.

[34] Rizwan Khan AY, Aziz Rana M, Naqvi DES, Asif A, Najeeb F, Naseem S. Health-seeking behaviour and its determinants following the COVID-19 pandemic. Cureus 2024; 16:e52225. 38347970 10.7759/cureus.52225 PMC10861314

[35] Otto SP, MacPherson A, Colijn C. Endemic does not mean constant as SARS-CoV-2 continues to evolve. Evolution 2024; 78:1092–108. 38459852 10.1093/evolut/qpae041

