# Supplementary material for: Changes to Endemic Respiratory Virus Circulation and Testing Before, During, and After the COVID-19 Pandemic
Source: Open Forum Infect Dis. 2025 Sep 26;12(9):ofaf493. doi: 10.1093/ofid/ofaf493 (PMC12464939; doi:10.1093/ofid/ofaf493)
Supplement: ofaf493_Supplementary_Data [file ofaf493_supplementary_data.zip › Supplementary Materials 1.3.docx]

**Supplemental Background - Ohio COVID-19 Mitigation Response:**Ohio was one of the earlier states to implement aggressive mitigation strategies, including a stay-at-home order in March 2020 and the closure of schools, non-essential businesses, and large gatherings. Over the course of 2020, the Ohio Department of Health issued a series of evolving health orders that mandated mask-wearing, social distancing, and capacity limits in public spaces and businesses. These orders were regularly updated as case trends fluctuated, with public messaging focusing heavily on community responsibility and voluntary compliance.

In 2021, Ohio transitioned toward vaccine distribution as the central pillar of its pandemic response. The state rolled out vaccines in phases, prioritizing frontline healthcare workers, elderly residents, and essential workers before eventually expanding eligibility to all residents aged 16 and older by March 2021. During this period, mass vaccination clinics were opened in urban centers, with mobile units extending access to underserved and rural populations. In tandem, the state began to revise restrictions—relaxing visitation rules in long-term care facilities, consolidating health orders into simplified guidance, and tying mask mandate removal to achieving specific case thresholds (e.g., fewer than 50 cases per 100,000 residents over two weeks).

By mid-2021, Ohio was focusing more on individual and community-level decision-making, with fewer blanket mandates and more targeted advisories. Travel advisories remained in place for high-transmission areas, and some restrictions persisted for indoor gatherings, though outdoor events were more leniently regulated. The state’s approach demonstrated a balance between public health caution and a gradual return to normalcy, placing increased trust in vaccination and local discretion as the pandemic progressed.

Compared to other states, Ohio’s COVID-19 response was generally moderate more proactive than some neighboring Midwestern states but less restrictive than states like California or New York. For example, Ohio implemented mask mandates and capacity restrictions relatively early but stopped short of the prolonged lockdowns or tiered reopening structures seen elsewhere. In contrast, states like Florida and Texas took more relaxed stances on mandates and restrictions, often resisting statewide mask orders altogether. Ohio’s phased vaccine rollout and clear communication on health metrics stood out as methodical and measured, positioning the state as a middle-ground model in the national landscape of COVID-19 policies

The Cleveland Department of Public Health (CDPH) implemented a multifaceted approach to managing the COVID-19 pandemic, emphasizing equitable access to resources and community engagement. In collaboration with organizations like MetroHealth and the Cuyahoga County Board of Health, CDPH conducted targeted vaccination drives, including efforts at homeless shelters to reach vulnerable populations. To bolster vaccination rates, initiatives such as the "Dollars for Doses" program provided financial incentives to residents receiving their first vaccine dose, particularly focusing on historically underserved neighborhoods.

Beyond vaccination efforts, CDPH partnered with Case Western Reserve University to enhance epidemiological tracking and data analysis, facilitating informed public health strategies. The department also prioritized the distribution of essential supplies—hand sanitizers, masks, and test kits—to community organizations, seniors, and daycare centers, ensuring widespread access to preventive tools.

**Supplemental methods:**

**Clinical testing platforms**: Similar to other institutions, respiratory viral panel testing platforms have changed dramatically throughout and after the pandemic due to supply chain restrictions and availability of new commercial assays. Each of the assays used had received FDA approval or emergency use authorization. Clinical testing platforms included the following: Xpert Xpress CoV-2 plus, Xpert Xpress Flu/RSV, Xpert Xpress CoV-2/Flu/RSV plus (Cepheid); Panther Aptima SARS-CoV-2, Panther Aptima SARS-CoV-2/Flu A/B, Panther Fusion Flu A/B/RSV, Panther Fusion SARS-CoV-2/Flu A/B/RSV (Hologic); cobas SARS-CoV-2, cobas SARS-CoV-2 & Influenza A/B (Roche); Cleveland Clinic SARS-CoV-2 assay; eSensor Respiratory Viral Panel (Genmark); NxTAG Respiratory Pathogen Panel Test (Luminex); Biofire Respiratory Panel 2.1 (bioMerieux).

**R and python open-source packages:** Analysis utilized SAS 9.4, R 4.4.1 and Python 3.10 with several open-source packages for analyses and visualizations.

The list of R packages and versions

- boot: 1.3-30
- datawizard: 0.12.3
- dplyr: 1.1.4
- dtplyr: 1.3.1
- ggplot2: 3.5.1
- haven: 2.5.4
- ISOweek: 0.6-2
- janitor: 2.2.0
- kableExtra: 1.4.0
- knitr: 1.48
- lubridate: 1.9.3
- patchwork: 1.2.0
- psych: 2.4.6.26
- quantreg: 5.98
- readxl: 1.4.3
- rlang: 1.1.4
- table1: 1.4.3
- tidyr: 1.3.1
- WaveletComp: 1.1

List of Python Packages used

- Matplotlib: 3.7.2
- numpy : 1.25.2
- pandas: 2.0.3
- python-dateutil: 2.8.2
- scipy: 1.11.2
- seaborn: 0.12.2

**Supplementary Tables:**

Supplementary Table 1: Yearly Change in Respiratory Virus Testing, Cleveland Clinic

| \| **Test Category** \| **2015** \| **2016** \| **2017** \| **2018** \| **2019** \| **2020** \| **2021** \| **2022** \| **2023** \| **2024** \| **Total** \| \| --- \| --- \| --- \| --- \| --- \| --- \| --- \| --- \| --- \| --- \| --- \| --- \| \| FLUA & FLUB \| 2715 \| 8668 \| 24832 \| 28686 \| 22188 \| 20436 \| 4141 \| 3958 \| 19 \| 0 \| 115643 \| \| FLUA & FLUB & RSV \| 0 \| 2647 \| 5044 \| 11579 \| 22331 \| 22275 \| 8460 \| 6100 \| 1794 \| 0 \| 80230 \| \| RSV \| 219 \| 978 \| 1497 \| 1491 \| 643 \| 1438 \| 492 \| 423 \| 26 \| 0 \| 7207 \| \| SARS-CoV-2 \| 0 \| 0 \| 0 \| 0 \| 0 \| 205151 \| 283954 \| 221772 \| 70310 \| 6478 \| 787665 \| \| SARS-CoV-2 & FLUA & FLUB \| 0 \| 0 \| 0 \| 0 \| 0 \| 776 \| 2344 \| 744 \| 515 \| 0 \| 4379 \| \| SARS-CoV-2 & FLUA & FLUB & RSV \| 0 \| 0 \| 0 \| 0 \| 0 \| 595 \| 40082 \| 110511 \| 126795 \| 65336 \| 343319 \| \|  \|  \|  \|  \|  \|  \|  \|  \|  \|  \|  \|  \| \| Expanded Panel * \| 3254 \| 4196 \| 5024 \| 3858 \| 4211 \| 5297 \| 3733 \| 5279 \| 2937 \| 0 \| 37789 \| \| Expanded Panel with SARS-CoV-2 ** \| 0 \| 0 \| 0 \| 0 \| 0 \| 8 \| 5795 \| 7487 \| 8271 \| 4735 \| 26296 \| \|  \|  \|  \|  \|  \|  \|  \|  \|  \|  \|  \|  \| \| Total \| 6188 \| 16489 \| 36397 \| 45614 \| 49373 \| 255976 \| 349001 \| 356274 \| 210667 \| 76549 \| 1402528 \| \| * includes FLUA, FLUB, RSV, PIV1-4, HMPV, BOCA, ADENO, RHINO/ENTERO, ENDEMIC CORONA \| \| \| \| \| \| \| \| \| \| \| \| \| ** includes SARS-CoV-2, FLUA, FLUB, RSV, PIV1-4, HMPV, BOCA, ADENO, RHINO/ENTERO, ENDEMIC CORONA \| \| \| \| \| \| \| \| \| \| \| \| |  |  |  |  |  |  |  |  |  |  |  |
| --- | --- | --- | --- | --- | --- | --- | --- | --- | --- | --- | --- | --- | --- | --- | --- | --- | --- | --- | --- | --- | --- | --- | --- | --- | --- | --- | --- | --- | --- | --- | --- | --- | --- | --- | --- | --- | --- | --- | --- | --- | --- | --- | --- | --- | --- | --- | --- | --- | --- | --- | --- | --- | --- | --- | --- | --- | --- | --- | --- | --- | --- | --- | --- | --- | --- | --- | --- | --- | --- | --- | --- | --- | --- | --- | --- | --- | --- | --- | --- | --- | --- | --- | --- | --- | --- | --- | --- | --- | --- | --- | --- | --- | --- | --- | --- | --- | --- | --- | --- | --- | --- | --- | --- | --- | --- | --- | --- | --- | --- | --- | --- | --- | --- | --- | --- | --- | --- | --- | --- | --- | --- | --- | --- | --- | --- | --- | --- | --- | --- | --- | --- | --- | --- | --- | --- | --- | --- | --- | --- | --- | --- | --- | --- | --- | --- | --- | --- | --- | --- | --- | --- | --- | --- | --- | --- | --- | --- | --- | --- | --- | --- | --- | --- | --- | --- | --- | --- | --- | --- | --- | --- | --- | --- | --- | --- | --- | --- | --- | --- |
|  |  |  |  |  |  |  |  |  |  |  |  |
|  |  |  |  |  |  |  |  |  |  |  |  |
|  |  |  |  |  |  |  |  |  |  |  |  |
|  |  |  |  |  |  |  |  |  |  |  |  |
|  |  |  |  |  |  |  |  |  |  |  |  |
|  |  |  |  |  |  |  |  |  |  |  |  |
|  |  |  |  |  |  |  |  |  |  |  |  |
|  |  |  |  |  |  |  |  |  |  |  |  |
|  |  |  |  |  |  |  |  |  |  |  |  |
|  |  |  |  |  |  |  |  |  |  |  |  |
|  | | | | | | | | | | | |
|  | | | | | | | | | | |  |

| Supplementary Table 2. Summary of peak positivity of endemic virus (Prepandemic: 12/29/2014 to 3/8/2020) and SARS-CoV-2 (Since outbreak: 3/9/2020–5/5/2024)   \| Factor \| FLUA (N=23,239) \| RSV (N=7,057) \| PIV3 (N=501) \| HMPV (N=782) \| SARS-CoV-2 (N=94,648) \| p-value \| \| --- \| --- \| --- \| --- \| --- \| --- \| --- \| \| Mode \| 7 \| 52 \| 24 \| 10 \| 50 \|  \| \| Mean \| 6 \| 52 \| 17 \| 10 \| 5 \|  \| \| ISO week*,  Median [P25, P75] \| 6  [3, 9] ^2,3,4,5^ \| 52  [49, 4] ^1,3,4,5^ \| 20  [14, 24] ^1,2,4,5^ \| 11  [4, 16] ^1,2,3,5^ \| 1  [48, 15] ^1,2,3,4^ \| *<.0001^b^* \| \| *A year was defined as spanning from Week 37 to Week 36 of the following year. p-values: ^b^=Kruskal-Wallis test.  ^1^: Significantly different from FLUA ^2^: Significantly different from RSV ^3^: Significantly different from PIV3 ^4^: Significantly different from HMPV ^5^: Significantly different from SARS-CoV-2 Post-hoc pairwise comparisons were done using Bonferroni adjustment. \| \| \| \| \| \| \|   Supplementary Table 3. Pairwise percent difference in peak positivity rate | | | |
| --- | --- | --- | --- | --- | --- | --- | --- | --- | --- | --- | --- | --- | --- | --- | --- | --- | --- | --- | --- | --- | --- | --- | --- | --- | --- | --- | --- | --- | --- | --- | --- | --- | --- | --- | --- | --- | --- | --- |
| Virus | Immediate Post- vs Pre-pandemic  (95% CI) | Post- vs Pre-pandemic (95% CI) | Post- vs Immediate Post-pandemic (95% CI) |
| FLUA | -7.6 (-8.1, -7.1) * | -9.0 (-9.2, -8.8) * | -1.4 (-2.0, -0.9) |
| RSV | -9.0 (-9.3, -8.7) * | -6.2 (-6.5, -6.0) * | 2.8 (2.5, 3.0) |
| PIV3 | -2.1 (-2.5, -1.6) | -0.2 (-0.4, 0.1) | 1.9 (1.5, 2.4) |
| HMPV | 0.7 (-0.7, 2.0) | -0.7 (-1.1, -0.4) | -1.4 (-2.7, -0.1) |
| ADENO | -1.3 (-2.1, -0.5) | 0.3 (0.0, 0.6) | 1.6 (0.8, 2.4) |
| RHINO/ENTERO | -8.3 (-10.0, -6.6) * | -1.6 (-2.2, -1.0) | 6.8 (5.1, 8.4) * |

Pre-pandemic period was defined as Dec 29, 2014 to Mar 8, 2020; immediate post-pandemic period as Mar 9, 2020 to May 10, 2020; post-pandemic period as May 11, 2020 to May 5, 2024

* Effect size and CIs fall beyond the margin (+/- 5%) in difference

Supplementary Table 4. Tabulated results of the interrupted time series analysis

| **Virus** | **Item** | **Estimate (95% CI)** | **p-value** |
| --- | --- | --- | --- |
| FLUA | Pre-pandemic slope | 1.6 (0.87, 2.3) | .0001 |
| FLUA | Post-pandemic slope | 1.1 (-0.52, 2.8) | .17 |
| FLUA | Slope change: post - pre | -0.44 (-2.2, 1.4) | .62 |
| FLUA | Immediate impact | -10 (-15, -5.3) | .0002 |
| RSV | Pre-pandemic slope | 0.31 (-0.41, 1.0) | .38 |
| RSV | Post-pandemic slope | -1.5 (-3.1, 0.13) | .071 |
| RSV | Slope change: post - pre | -1.8 (-3.5, -0.048) | .044 |
| RSV | Immediate impact | -0.72 (-5.6, 4.1) | .76 |
| PIV3 | Pre-pandemic slope | -0.12 (-0.41, 0.17) | .40 |
| PIV3 | Post-pandemic slope | -0.63 (-1.4, 0.18) | .12 |
| PIV3 | Slope change: post - pre | -0.51 (-1.4, 0.36) | .24 |
| PIV3 | Immediate impact | 1.8 (-0.37, 3.9) | .10 |
| HMPV | Pre-pandemic slope | 0.0088 (-0.43, 0.45) | .97 |
| HMPV | Post-pandemic slope | 0.31 (-0.67, 1.3) | .52 |
| HMPV | Slope change: post - pre | 0.30 (-0.76, 1.4) | .57 |
| HMPV | Immediate impact | -0.89 (-3.8, 2.0) | .54 |
| ADENO | Pre-pandemic slope | -0.56 (-0.72, -0.40) | <.0001 |
| ADENO | Post-pandemic slope | 1.0 (0.28, 1.7) | .0085 |
| ADENO | Slope change: post - pre | 1.6 (0.80, 2.4) | .0003 |
| ADENO | Immediate impact | -0.0053 (-1.7, 1.7) | >.99 |
| RHINO/ENTERO | Pre-pandemic slope | 0.32 (-0.44, 1.1) | .40 |
| RHINO/ENTERO | Post-pandemic slope | 0.81 (-0.45, 2.1) | .20 |
| RHINO/ENTERO | Slope change: post - pre | 0.49 (-1.1, 2.0) | .52 |
| RHINO/ENTERO | Immediate impact | -4.2 (-7.6, -0.75) | .019 |
