# Supplementary figures and images for: Changes to Endemic Respiratory Virus Circulation and Testing Before, During, and After the COVID-19 Pandemic
Source: Open Forum Infect Dis. 2025 Sep 26;12(9):ofaf493. doi: 10.1093/ofid/ofaf493 (PMC12464939; doi:10.1093/ofid/ofaf493)

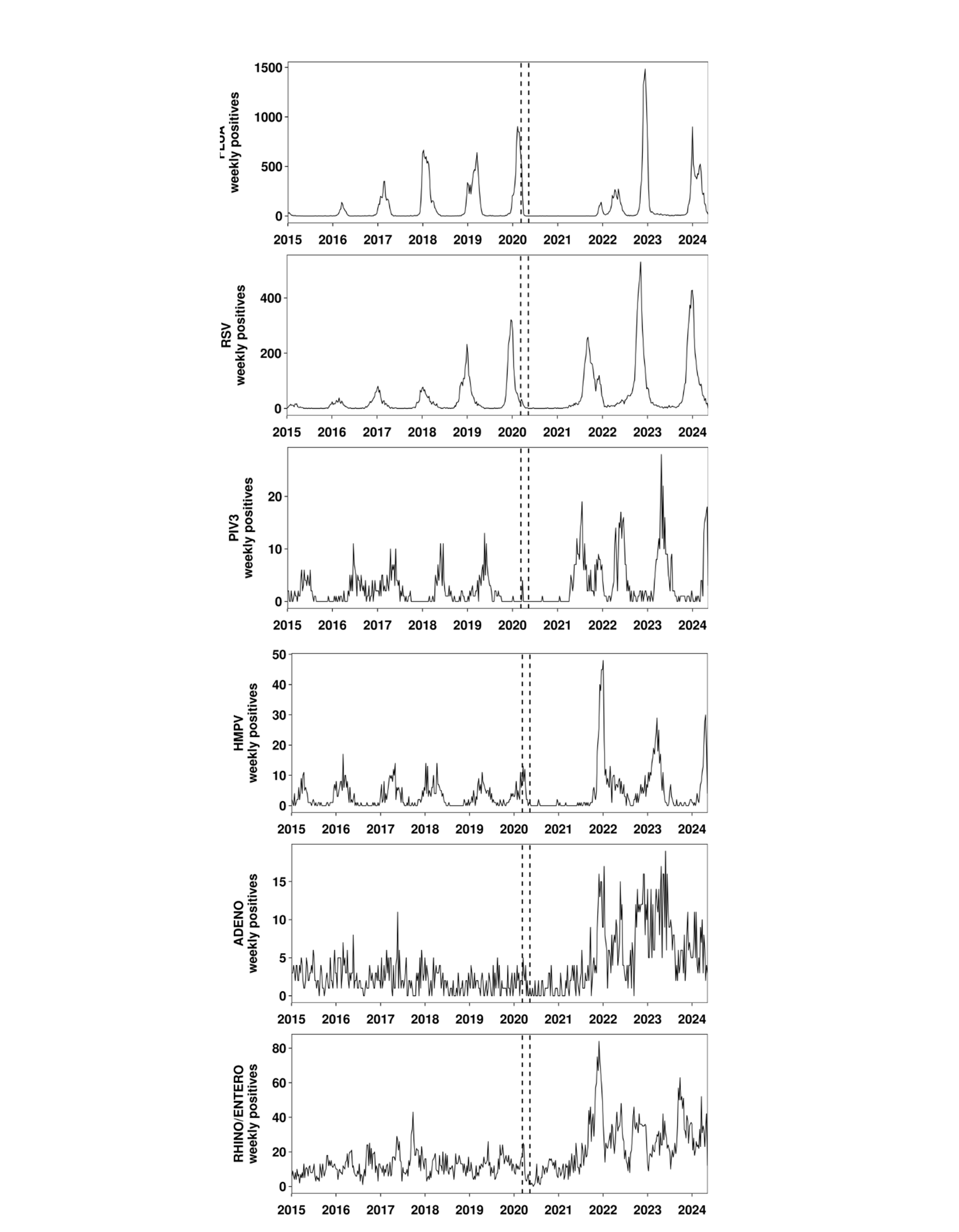

Supplement: ofaf493_Supplementary_Data [file ofaf493_supplementary_data.zip › Supplementary figure 1.2.tif]
